# Supplementary material for: Vanoxerine kills mycobacteria through membrane depolarization and efflux inhibition
Source: Front Microbiol. 2023 Jan 26;14:1112491. doi: 10.3389/fmicb.2023.1112491 (PMC9909702; doi:10.3389/fmicb.2023.1112491)
Supplement: Supplementary file 1 [file Table_1.DOCX]

Supplementary Table 1: Table of Bacterial Strains used in the study

| Microorganism | Strain | Plasmid/Mutant | Antibiotic Resistance | Reference |
| --- | --- | --- | --- | --- |
| Mycobacterium smegmatis | mc^2^155 |  |  |  |
| Mycobacterium smegmatis | mc^2^155 | pVV16 (empty) | Kanamycin | This study |
| Mycobacterium smegmatis | mc^2^155 | pVV16-MtAroB | Kanamycin | This study |
| Mycobacterium smegmatis | mc^2^155 | pTIC6a (empty) | Kanamycin | This study |
| Mycobacterium smegmatis | mc^2^155 | pTIC6a-MtAroB | Kanamycin | This study |
| Mycobacterium bovis | BCG Pasteur |  |  |  |
| Mycobacterium bovis | BCG Pasteur | ∆recG |  | (Batt et al., 2015) |
| Escherichia coli | BL21 (DE3) | pET28a-MtAroB | Kanamycin | This study |
| Corynebacterium glutamicum | 13032 |  |  |  |
| Corynebacterium glutamicum | 13032 | ∆pks |  | (Gande et al., 2004) |
| Enterococcus faecium | 64/3 |  | Rifampicin, fusidic acid | (Werner et al., 2003) |
| Staphylococcus aureus | SA01 |  |  |  |
| Klebsiella pneumoniae | Ecl08 (KP02) |  |  |  |
| Acinetobacter baumanii | AYE (AC05) |  |  |  |
| Pseudomonas aeruginosa | PA14 |  |  |  |

**Supplementary Material References**

Batt, S.M., Cacho Izquierdo, M., Castro Pichel, J., et al. (2015) Whole Cell Target Engagement Identifies Novel Inhibitors of *Mycobacterium tuberculosis* Decaprenylphosphoryl-β- d -ribose Oxidase. *ACS Infectious Diseases*, 1 (12): 615–626. doi:10.1021/acsinfecdis.5b00065.

Gande, R., Gibson, K.J.C., Brown, A.K., et al. (2004) Acyl-CoA Carboxylases (accD2 and accD3), Together with a Unique Polyketide Synthase (Cg-pks), Are Key to Mycolic Acid Biosynthesis in Corynebacterianeae Such as Corynebacterium glutamicum and Mycobacterium tuberculosis. *Journal of Biological Chemistry*, 279 (43): 44847–44857. doi:10.1074/jbc.M408648200.

Werner, G., Willems, R.J.L., Hildebrandt, B., et al. (2003) Influence of Transferable Genetic Determinants on the Outcome of Typing Methods Commonly Used for Enterococcus faecium. *Journal of Clinical Microbiology*, 41 (4): 1499–1506. doi:10.1128/JCM.41.4.1499-1506.2003.
