# Supplementary material for: Vanoxerine kills mycobacteria through membrane depolarization and efflux inhibition
Source: Front Microbiol. 2023 Jan 26;14:1112491. doi: 10.3389/fmicb.2023.1112491 (PMC9909702; doi:10.3389/fmicb.2023.1112491)
Supplement: Supplementary file 5 [file Table_5.DOCX]

Supplementary Table 5: Significantly dysregulated genes comparing treatment of M. bovis BCG following treatment of 15 and 30 µg/ml of vanoxerine. Data displayed in Supplementary Figure 6.

| ID | ID in TB | Functional Annotation | log2FoldChange | padj |
| --- | --- | --- | --- | --- |
| acpM | acpM | Mycolic acid biosynthesis | -1.7106273 | 4.13E-24 |
| kasA | kasA | Mycolic acid biosynthesis | -1.6430428 | 4.24E-43 |
| fabD | fabD | Mycolic acid biosynthesis | -1.5780505 | 6.23E-54 |
| kasB | kasB | Mycolic acid biosynthesis | -1.3809422 | 6.23E-68 |
| BCG_2265 | Unknown | Unknown | -1.118137 | 3.46E-11 |
| ahpE | ahpE | Peroxidase | -1.0634044 | 4.20E-12 |
| cysK2 | cysK2 | Cysteine synthesis | 1.01017264 | 1.15E-28 |
| lat | lat | Lysine - glutamate conversion | 1.04721741 | 6.53E-28 |
| PPE47 | PPE47/PPE48 (Rv3021c) | Unknown | 1.05591478 | 1.01E-13 |
| esxR | esxR | Secreted protein | 1.06344389 | 1.09E-05 |
| PPE46 | PPE46 (Rv3018c) | Unknown | 1.06624228 | 1.89E-44 |
| BCG_0231 | Rv0194 | Multidrug efflux ATP-binding/permease protein | 1.10345536 | 4.80E-96 |
| BCG_0728c | Rv0679c | Unknown | 1.15139373 | 5.48E-98 |
| BCG_3926 | Rv3863 | Unknown | 1.17968576 | 3.14E-39 |
| IS1606' | Rv0850 | Transposase | 1.19977257 | 6.15E-06 |
| BCG_1993c | Unknown | Unknown (DUF402) | 1.22347787 | 4.71E-59 |
| mmpL5 | mmpL5 | Efflux | 1.25195902 | 5.07E-87 |
| lpqS | lpqS | Lipoprotein of unknown function | 1.26228081 | 1.46E-22 |
| echA5 | echA5 | Fatty acid oxidation | 1.27325471 | 2.22E-53 |
| PPE32 | PPE32 | Unknown | 1.32645124 | 4.02E-28 |
| BCG_0369c | eccE3 (Rv0292) | ESX-3 secretion system protein EccE | 1.32867714 | 4.13E-120 |
| PPE33a | PPE33a | Unknown | 1.35099382 | 2.60E-11 |
| BCG_0842c | Rv0789c | Unknown | 1.3900481 | 6.68E-89 |
| mmpS5 | mmpS5 | Efflux | 1.4394337 | 1.95E-25 |
| BCG_1466c | Rv1405 | Putative methyltransferase | 1.45830259 | 3.67E-14 |
| hsp | hsp | Stress response | 1.53579295 | 4.00E-158 |
| BCG_0368c | Rv0329c | Putative methyltransferase | 1.53961684 | 2.22E-31 |
| BCG_3766c | Rv3706 | Unknown | 1.82697662 | 1.61E-22 |
| BCG_0365 | Rv0325 | Putative methyltransferase | 1.84468403 | 1.96E-44 |
| BCG_0367 | Rv0328 | Possible transcriptional regulator | 2.09420983 | 1.04E-105 |
| cyp135A1 | cyp135A1 | Cytochrome P450 | 2.42351955 | 7.71E-73 |
