# Supplementary material for: Vanoxerine kills mycobacteria through membrane depolarization and efflux inhibition
Source: Front Microbiol. 2023 Jan 26;14:1112491. doi: 10.3389/fmicb.2023.1112491 (PMC9909702; doi:10.3389/fmicb.2023.1112491)
Supplement: Supplementary file 6 [file Data_Sheet_1.PDF]

Supplementary Material

Supplementary Figures

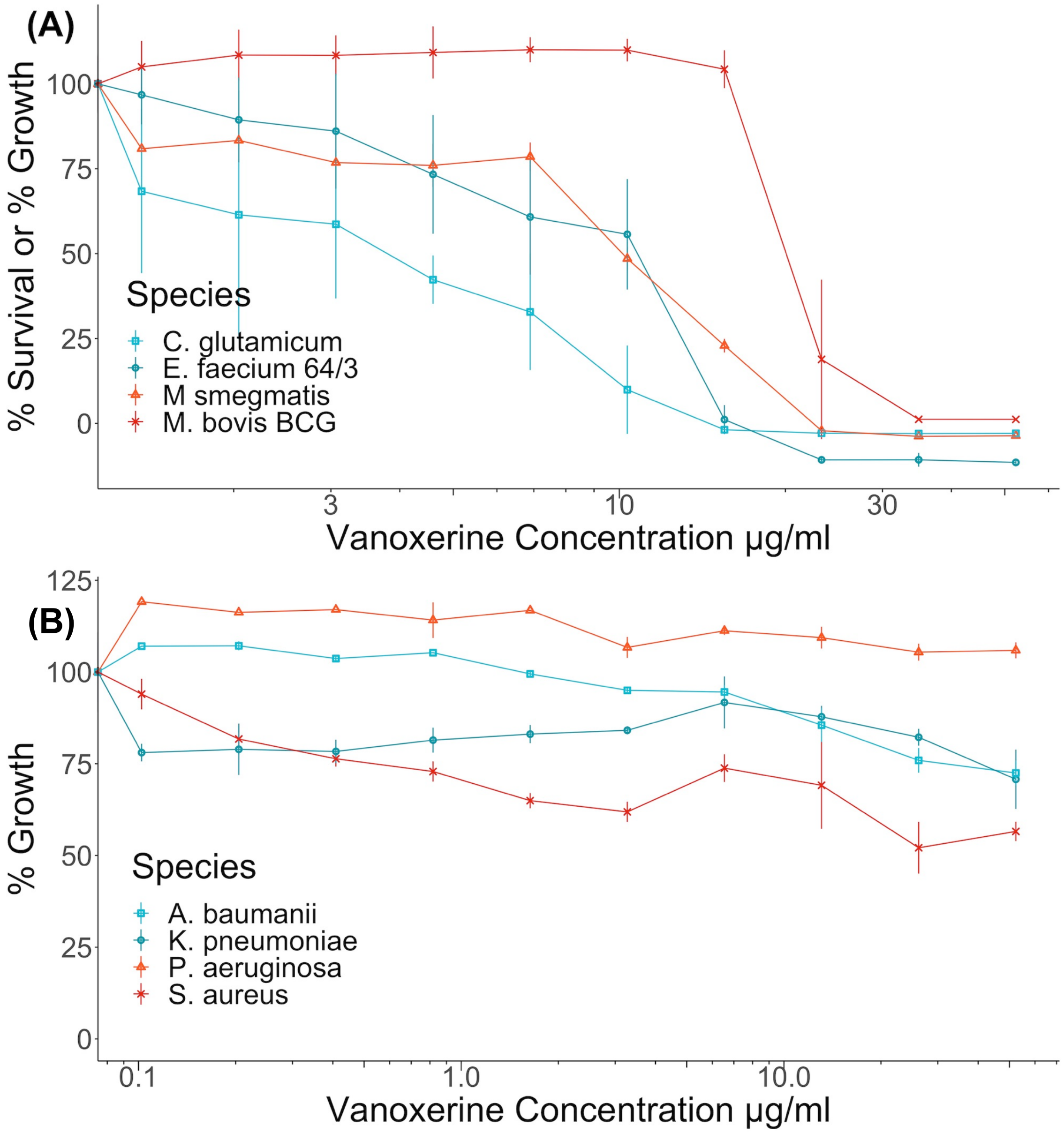

Supplementary Figure 1: % Survival or % Growth curves of all tested species against Vanoxerine. (A) Species vanoxerine inhibited. (B) Species vanoxerine did not inhibit. Bacterial cultures were diluted to OD_{600} = 0.05. A 2:3 serial dilution (A) or a 1:2 serial dilution (B) of vanoxerine was performed and the diluted cell cultures were added. The plates were incubated for 21 h (*M. smegmatis*), 23 h (*C. glutamicum*), 24 h (*E. faecium* 64/3*, A. baumanii, K. pneumoniae, P. aeruginosa & S. aureus*) or 6 days (*M. bovis* BCG). For *E. faecium* 64/3*, A. baumanii, K. pneumoniae, P. aeruginosa & S. aureus*, the OD_{600} was measured. For *M. smegmatis, C. glutamicum* and *M. bovis* BCG, resazurin was added and the plates re-incubated for 1 h (*C. glutamicum*), 3 h (*M. smegmatis*) or 24 h (*M. bovis* BCG). Then the resorufin fluorescence was measured. In all cases, the % survival was calculated relative to a positive control (e.g., Rifampicin) and a negative control (DMSO only).

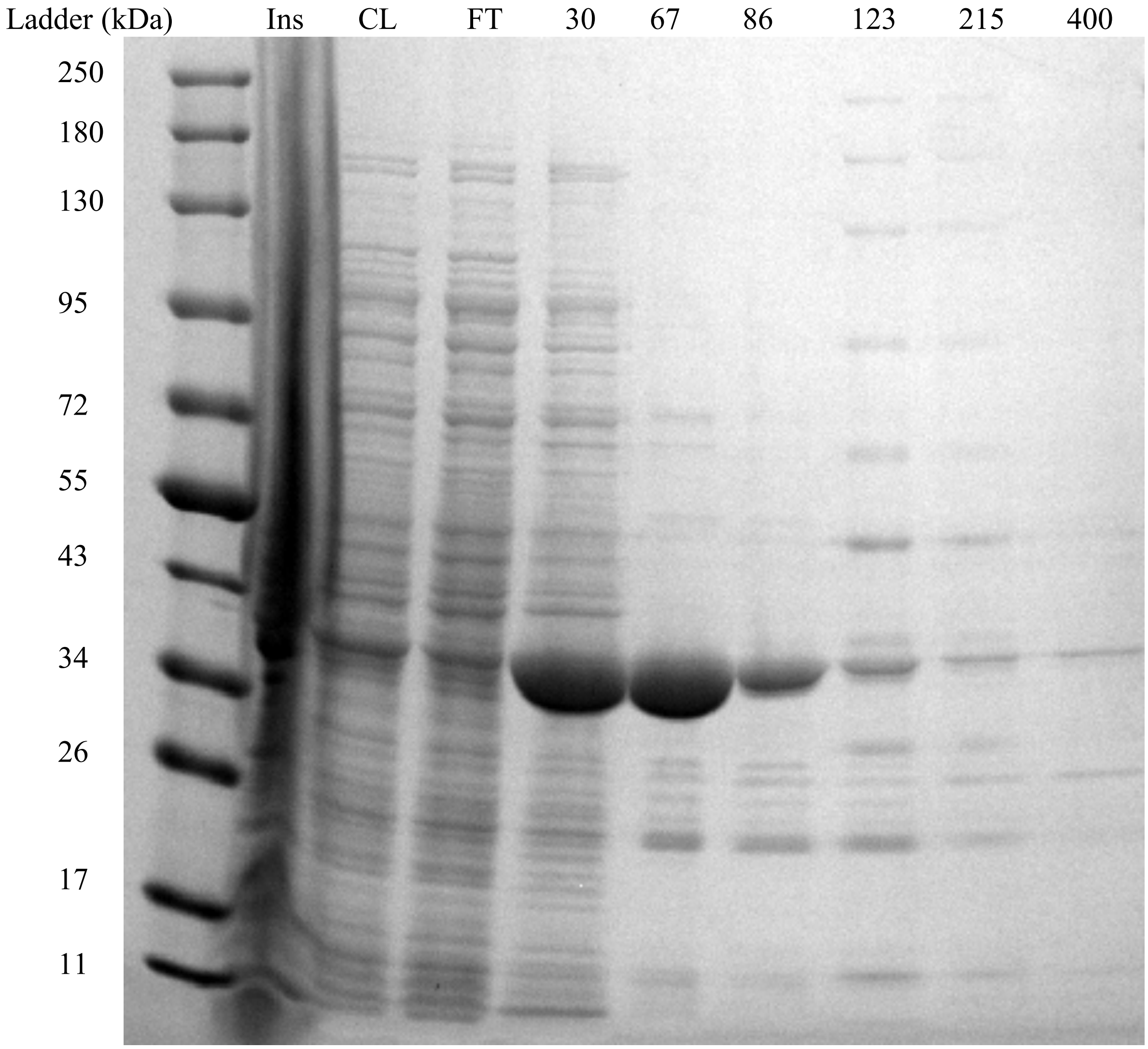

Supplementary Figure 2: Mt-AroB (39 kDa) was successfully purified using a nickel NTA column. *E. coli* BL21(DE3) cells containing the pET28a-MtAroB plasmid, had protein expression induced using IPTG for 18 hours. The cells were harvested, resuspended in buffer and lysed. This lysate was clarified, before being loaded onto a nickel NTA column. The column was washed with buffer containing 30 mM imidazole, before the protein was eluted, using increasing imidazole concentrations. This SDS-PAGE gel displays samples of each stage of purification, with MtAroB appearing at 39 kDa. Ins = insoluble fraction, CL = clarified lysate, FT = flow-through, 30 to 400 represent mM concentrations of imidazole in the buffer.

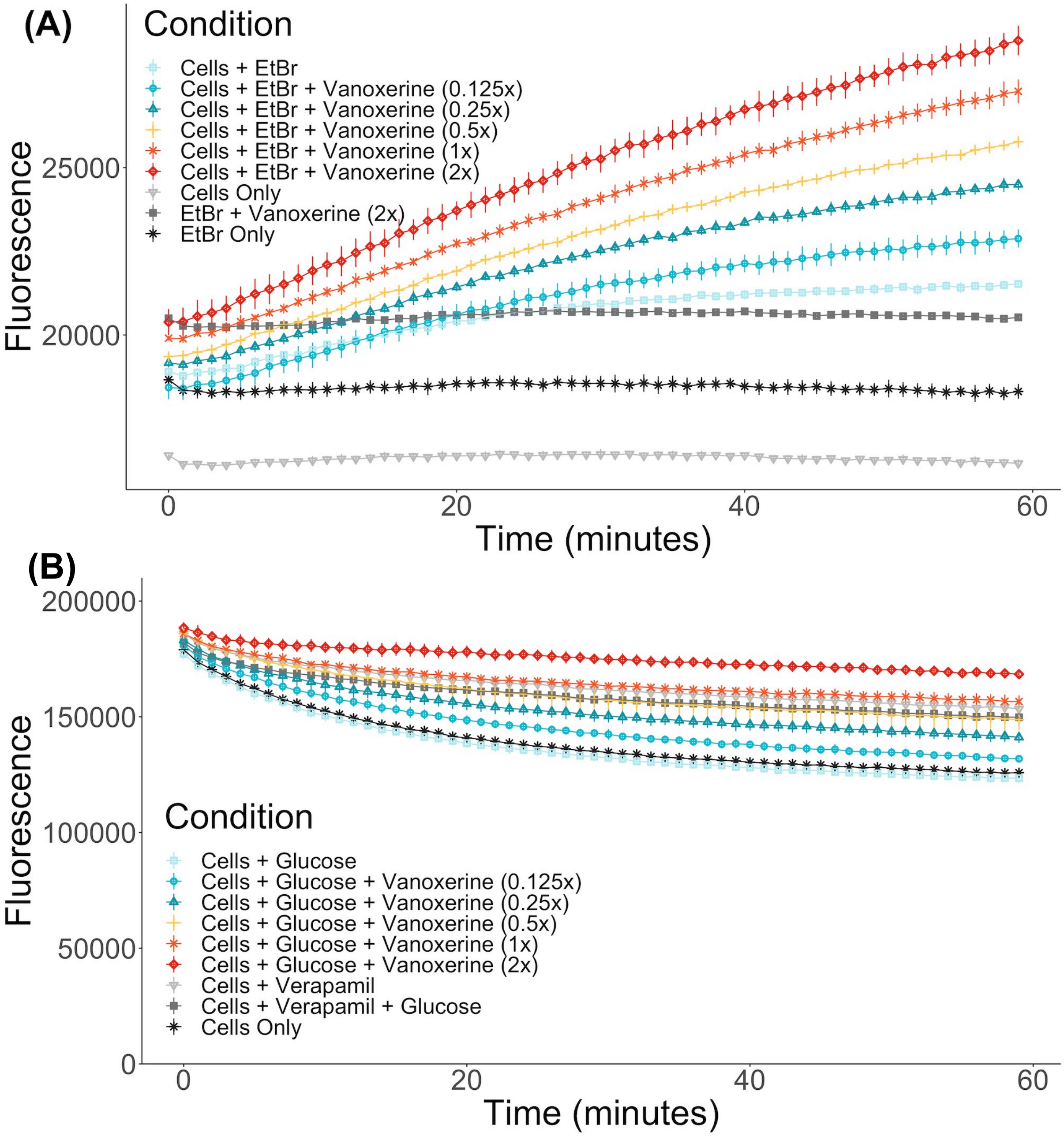

Supplementary Figure 3: (A) Vanoxerine treated *M. bovis* BCG accumulated ethidium bromide at an increased rate. *M. bovis* BCG was washed and re-suspended in PBS + 0.05% tween-80 + 0.4% glucose + 0.625 µg/ml ethidium bromide. The cells were immediately added to a 96-well plate containing varying vanoxerine concentrations and the fluorescence was measured for 1 hour. Vanoxerine MIC_{99} = 26 µg/ml. N=3. **(B) *M. bovis* BCG efflux of ethidium bromide was inhibited by sub-inhibitory concentrations of vanoxerine.** *M. bovis* BCG was allowed to accumulate ethidium bromide (0.625 µg/ml) for 1 hour, using verapamil (50 µg/ml). Then the cells were resuspended in fresh PBS + 0.05% tween-80. Cells were either added directly or mixed with glucose (0.4%) before adding to a 96-well plate containing vanoxerine or verapamil. Vanoxerine MIC_{99} = 26 µg/ml. N=3

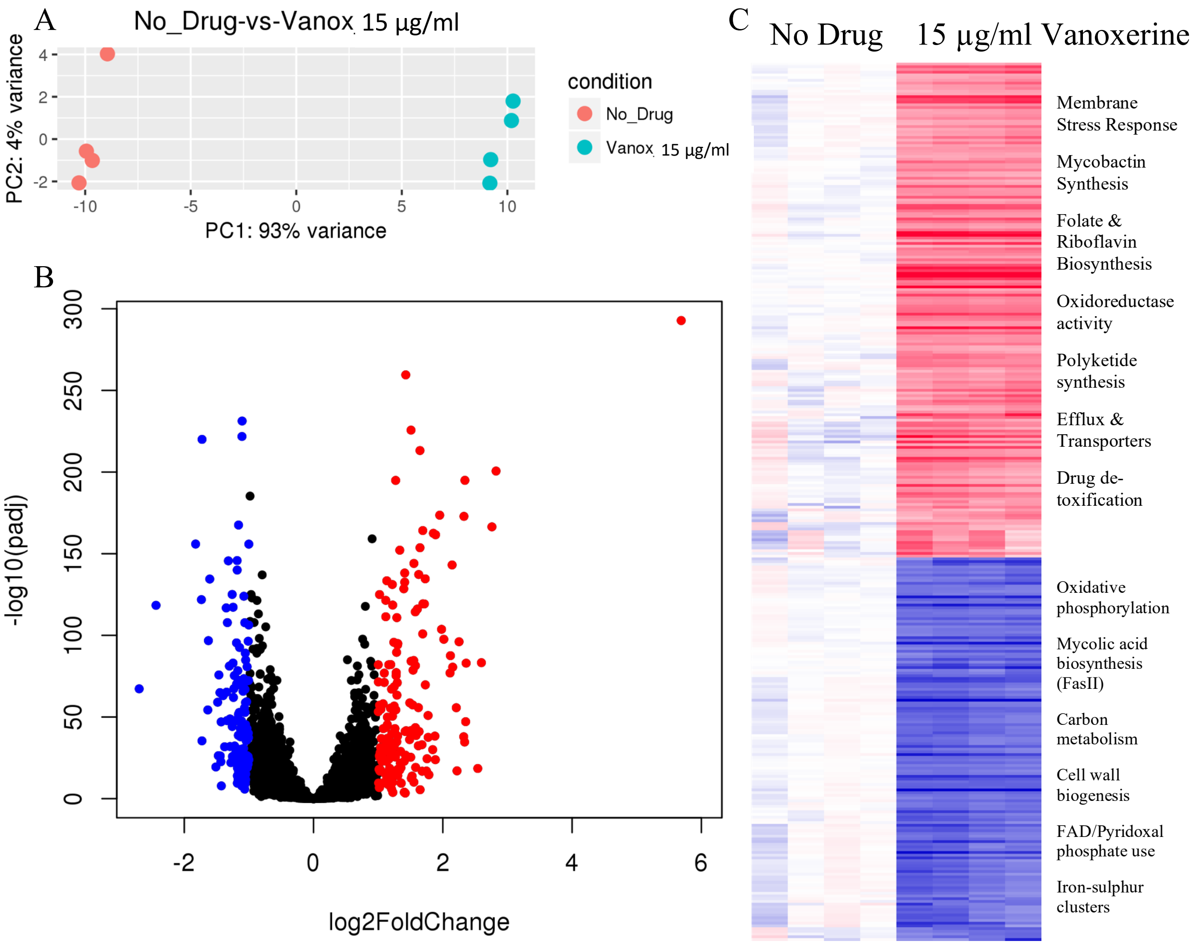

Supplementary Figure 4: Transcriptomic changes following drug treatment of *M. bovis* BCG relative to a DMSO only control. RNA sequencing was performed on four samples from each condition. (A) Principal component analysis highlighting the difference in transcriptional variation between DMSO-only controls and 15 µg/ml vanoxerine, following 8-hours of drug treatment. (B) Volcano plot indicating the significantly dysregulated genes. Highlighted points have a fold change >2 and a p-adjusted value of <0.05; red = up-regulated, blue = down-regulated. (C) Clustered comparison of transcriptional differences between DMSO-only and 15 µg/ml vanoxerine. Biological functions of the dysregulated genes are highlighted

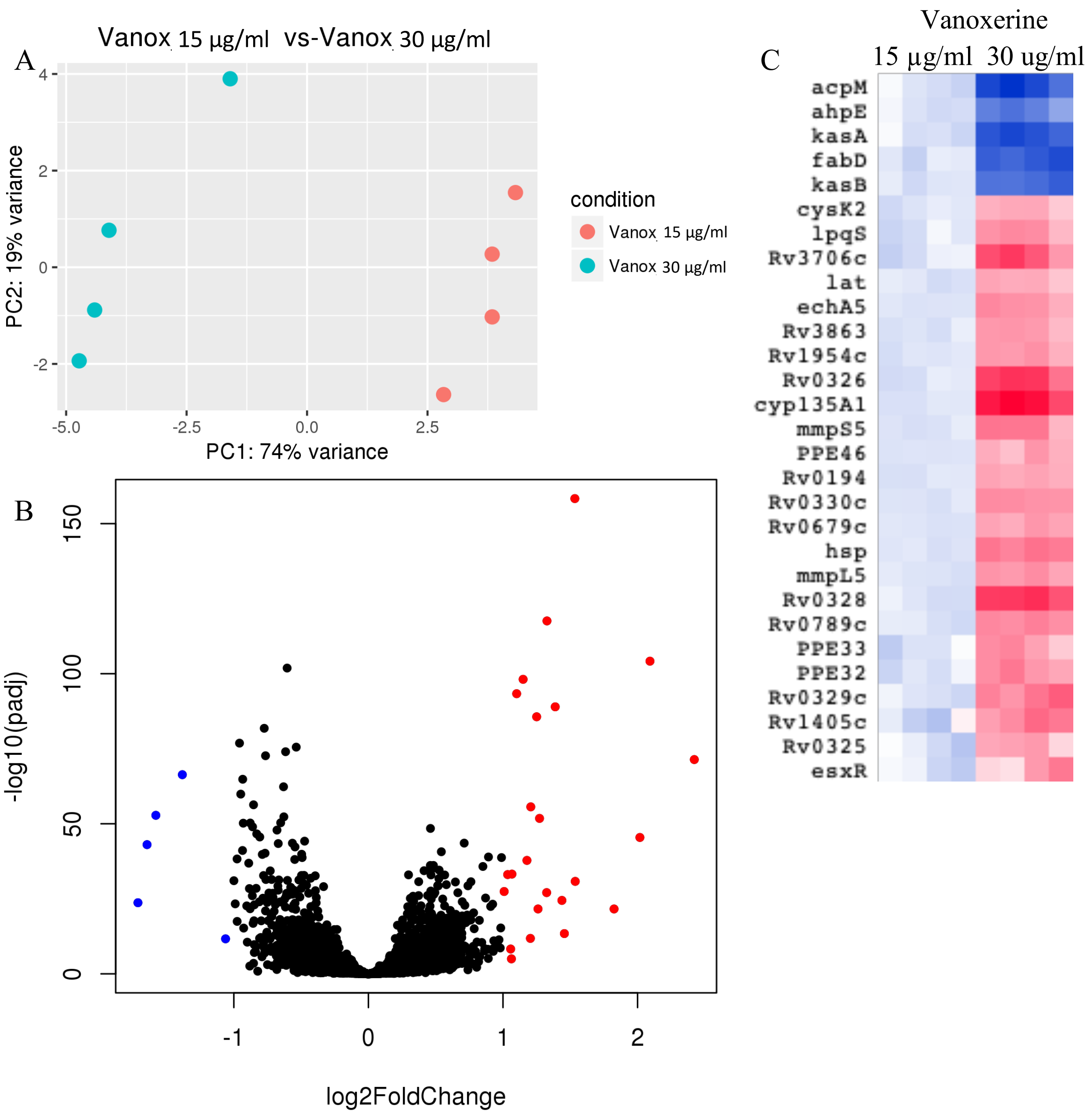

Supplementary Figure 5: Transcriptomic changes following drug treatment of *M. bovis* BCG, comparing 15 to 30 µg/ml vanoxerine. RNA sequencing was performed on four samples from each condition. (A) Principal component analysis highlighting the difference in transcriptional variation between 15 and 30 µg/ml of vanoxerine, following 8-hours of drug treatment. (B) Volcano plot indicating the significantly dysregulated genes. Highlighted points have a fold change > 2 and a p-adjusted value of <0.05; red = up-regulated, blue = down-regulated. (C) Clustered comparison of transcriptional differences between 15 and 30 µg/ml of vanoxerine. Dysregulated gene transcripts are named (equivalent tuberculosis naming is used if known).

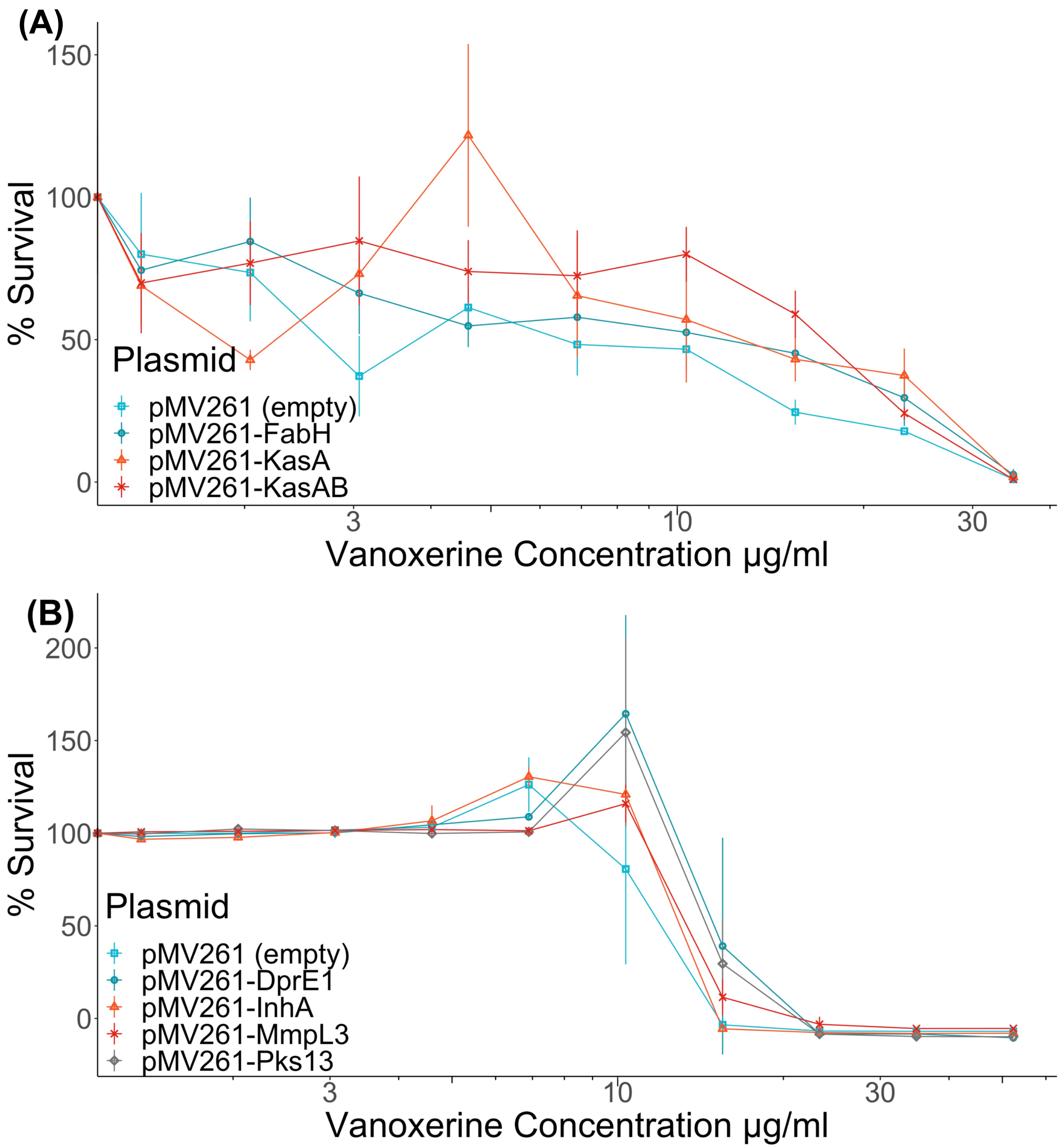

Supplementary Figure 6: Mycobacteria over-expressing genes from the mycolic acid biosynthetic pathway had no impact on vanoxerine resistance. (A) *M. smegmatis* (B) *M. bovis* BCG. Bacterial cultures were diluted to OD_{600} = 0.05. A 2:3 serial dilution of vanoxerine was performed into DMSO, transferred to an assay plate (1 µl per well) and the diluted cell cultures was added (99 µl). The plates were incubated at 37 ˚C, for 21 h (*M. smegmatis*) or 6 days (*M. bovis* BCG). For *M. smegmatis* and *M. bovis* BCG, resazurin was added and the plates re-incubated for 3 h (*M. smegmatis*) or 24 h (*M. bovis* BCG). Then the resorufin fluorescence was measured. The %survival was calculated relative to a positive control and a negative control.
